# Identification and Quantification of Oxidoselina-1,3,7(11)-Trien-8-One and Cyanidin-3-Glucoside as One of the Major Volatile and Non-Volatile Low-Molecular-Weight Constituents in Pitanga Pulp

**DOI:** 10.1371/journal.pone.0138809

**Published:** 2015-09-22

**Authors:** Denise Josino Soares, Marc Pignitter, Miriam Margit Ehrnhöfer-Ressler, Jessica Walker, Isabella Montenegro Brasil, Veronika Somoza

**Affiliations:** 1 CAPES Foundation, Ministry of Education of Brazil, Brasília, Brazil; 2 Department of Nutritional and Physiological Chemistry, Faculty of Chemistry, University of Vienna, Vienna, Austria; 3 Department of Food Technology, Federal University of Ceara, Fortaleza, Brazil; The University of Tokyo, JAPAN

## Abstract

The pulp of pitanga (*Eugenia uniflora* L.) is used to prepare pitanga juice. However, there are no reports on the identification and quantification of the main constituents in pitanga pulp. The aim of this study was to identify and quantify the major volatile and non-volatile low-molecular-weight constituents of the pulp. Isolation of volatile compounds was performed by solvent-assisted flavor evaporation technique. Characterization of the main volatile and non-volatile constituents was performed by GC-MS, LC-MS and NMR spectroscopy. For quantitative measurements, the main volatile compound needed to be isolated from pitanga pulp to obtain a commercially not available reference standard. Cyanidin-3-glucoside was determined as one of the most abundant non-volatile pulp compound yielding 53.8% of the sum of the intensities of all ions detected by LC-MS. Quantification of cyanidin-3-glucoside in pitanga pulp resulted in a concentration of 344 ± 66.4 μg/mL corresponding to 688 ± 133 μg/g dried pulp and 530 ± 102 μg/g fruit. For the volatile fraction, oxidoselina-1,3,7(11)-trien-8-one was identified as the main volatile pulp constituent (27.7% of the sum of the intensities of all ions detected by GC-MS), reaching a concentration of 89.0 ± 16.9 μg/mL corresponding to 1.34 ± 0.25 μg/g fresh pulp and 1.03 ± 0.19 μg/g fruit. The results provide quantitative evidence for the occurrence of an anthocyanin and an oxygenated sesquiterpene as one of the major volatile and non-volatile low-molecular-weight compounds in pitanga pulp.

## Introduction

Pitanga (*Eugenia uniflora* L.), which belongs to the family of Myrtaceae, is a tropical tree originating from South America [1]. The pitanga tree is mainly cultivated in Argentina, Brazil, Paraguay and Uruguay. Due to its high palatability, especially the pitanga fruit is used commercially by Brazilian industry with a sales volume of approximately 1700 tons per year [2]. The fruit pulp, which corresponds to approximately 77% of the whole fruit, is used for commercialization. Although pitanga is a versatile raw material, the juice is one of the main products that is prepared from the pulp of the fruit. Pitanga juice is a non-fermented beverage, which is characterized by a pulp content of 350 g/kg and a glucose content of 71 g/kg in tap water, according to Brazilian law.

Pitanga is gaining popularity worldwide especially in the European, Indian and North American markets [2]. Its worldwide distribution is not only driven by the palatability of the fruit, but also by its low content in lipids (4.0–8.8 g/kg) [1], its low caloric content (430–510 cal/kg) [1] and high amounts of polyphenols (58.1 g/kg ferulic acid equivalents in dried fruits for the purple variety) [3] and carotenoids (12000–20000 I.U. carotene/kg) [1]. The chemical composition of pitanga leaves and fruits differs widely, and is influenced by the environmental harvesting conditions, the developmental stage and the genotype [4]. There are three abundant pitanga varieties [1]. These are the orange-, red- and purple-fleshed pitanga fruits, with the latter two being the most common ones. Each of the pitanga varieties can be classified into different developmental stages, such as the green (immature), the red to the purple (mature) stage [3]. Celli *et al*. [3] determined a higher content of 320 ± 1.84 μg/g aglycone equivalents of myricetin 3-O-rhamnoside in early developmental (green) stage of purple-fleshed pitanga compared to the ripe (purple) pitanga from purple variety containing only 280 ± 1.37 μg/g aglycone equivalents of myricetin 3-O-rhamnoside.

Differences in the chemical composition of pitanga fruit chiefly determine its biological activity. Celli *et al*. [3] could show that fruits from purple variety at the early developmental (green) stage were associated with the highest antioxidant activity, as evidenced by an enhanced DPPH^●^ scavenging activity of the green pitanga pulp, reaching 74.9 ± 0.8% compared to the DPPH^●^ scavenging activity of the red (ripe) pitanga pulp reaching solely 41.9 ± 1.1%.

Besides its antioxidant activity, pitanga leaf extracts but not fruits were shown to exert beneficial effects in animal studies. It was reported that the pitanga extracts possess anti-inflammatory effects evaluated by inhibition of carrageenan-induced paw oedema in rats [5]. Antihypertensive effects of pitanga leaf extracts were shown after intraperitoneal injection of 6 mg aqueous crude extract of dried pitanga leaves per kilogram body weight, which decreased blood pressure of normotensive rats by 47.1 ± 8.2% compared to untreated normotensive rats [6]. The same study also revealed a vasodilating activity of 12 g/L dried pitanga leaves by a decrease of the perfusion pressure of rat hindquarters by 32.3 ± 11.5%. An inhibitory effect of ethanolic extracts of pitanga leaves on the increase in plasma glucose and plasma triglyceride level in mice was revealed by an oral glucose tolerance test and an oral corn oil tolerance test [7]. Gastrointestinal motility-enhancing effects of pitanga leaf extract (0.2%) were shown by the contractile responses of the rat duodenum, which were within the 50% maximal response of acetylcholine (4.4–70 x 10^−7^ M) [8]. Pitanga leaf extracts were also demonstrated to exert antimicrobial and antifungal effects [9,10]. Recently, pitanga juice was shown, for the first time, to exploit an anti-inflammatory effect on gingival epithelial cells by attenuating the interleukin-8 release by 55 ± 8.2% and 52 ± 11% in non-stimulated and *lipopolysaccharides*-stimulated cells, respectively [11].

Identification of compounds responsible for these pharmacological effects is of tremendous interest. Even though there is evidence underlining the beneficial health effects of the pitanga leaf extracts, reports on the bioactivity of pitanga fruit are limited. Also, the pulp, which is used to prepare the juice, has not yet been chemically characterized. The current study identified and quantified the major volatile but also non-volatile low-molecular-weight constituents in pitanga pulp from the purple variety.

## Experimental

### Materials and chemicals

Cyanidin-3-glucoside was purchased from Polyphenols Laboratories AS (Sandnes, Norway). Original Brazilian pitanga fruits of the purple variety (Eugenia uniflora L., Myrtaceae) with eight years of age were harvested at Fortaleza farm located in the city of Gandu, Bahia, Brazil at 13 44'S, 39 28'W by the Brazilian Agricultural Research Corporation (Embrapa). The owner of the land gave permission to collect pitanga fruits. We confirm that the field studies did not involve endangered or protected species being commonly grown by local farmers in these regions. A total amount of three kilograms of ripe pitanga was harvested in October 2011. To obtain the pulp, undamaged fruits were selected and sanitized. The edible portion of the fruits was processed in a semi-industrial depulper. The pulp was stored in sealed polyethylene bags, frozen in liquid nitrogen and transferred to Vienna, Austria, in a polystyrene box containing dry ice. Upon arrival the pulp was stored at -80°C until analysis. All other chemicals were ordered from Sigma Aldrich (Vienna, Austria) or Carl Roth (Karlsruhe, Germany).

### Identification and quantification of cyanidin-3-glucoside

For identification of cyanidin-3-glucoside, pitanga pulp was freeze-dried and extracted based on the method from Celli *et al*. [3]. Briefly, five grams of dried pitanga pulp were stirred with 10 mL of acetone/water/acetic acid (70:29:1, v/v/v) for just two minutes to ensure stability of cyanidin-3-glucoside. The pulp extract was centrifuged at 4500 *g* for 1 min at 4°C, and passed through a polyvinylidene fluoride syringe filter with membrane pore size of 0.45 μm and a nominal diameter of 13 mm. The filtrate (5 μL) was injected into an HPLC system (Shimadzu Prominence, Vienna, Austria), coupled to a Shimadzu LC-MS-2020 single quadrupole mass spectrometer (Vienna, Austria). The samples were separated at 25°C on a Luna C18 column (250 x 3.00 mm, 5 μm, 100 Å, Phenomenex, Aschaffenburg, Germany) using a mobile phase consisting of 0.3% (v/v) formic acid in water (A) and 0.3% (v/v) formic acid in methanol (B) with a flow rate of 0.5 mL/min. A linear gradient was applied starting with 5% B and 95% A. After isocratic conditions for 1.5 min the solvent B reached 10% at 4 min. After 10 min the solvent B achieved 15%. Prior to returning to the initial conditions, solvent B reached 50% after a total of 35 min. To identify the major non-volatile component in pitanga pulp, the mass spectrometer was operated in the ESI (+) mode scanning m/z 100–1000. For MS analysis, a nebulizing gas (N_2_) flow of 1.5 L/min, a drying gas (N_2_) flow of 5.0 L/min, a desolvation line temperature of 250°C, a heat block temperature of 200°C and an interface voltage of 4.5 kV were selected. In-source fragmentation and the standard, cyanidin-3-glucoside chloride, were used to identify cyanidin-3-glucoside. In-source fragmentation was achieved by setting the desolvation line voltage at 20 V and the Qarray DC voltage at 40 V. Fragmentation of cyanidin-3-glucoside resulted in the formation of the deglucosylated product ion with m/z 287 for cyanidin [12]. For quantification of cyanidin-3-glucoside by external calibration, selected ion monitoring was used at m/z 449. Cyanidin-3-glucoside chloride was used for a five-point calibration curve in the range of 10–400 μg/mL. The linearity of the curve was confirmed by the regression coefficient R^2^ = 0.999. The LOD and LOQ were determined to be 41.1 ± 13.3 ng/mL and 137 ± 44.3 ng/mL, respectively, according to the approach described in the European Pharmacopoeia. The results were expressed as μg/mL of pulp.

### Isolation of volatile compounds from pitanga pulp

A total of 100 g of pitanga pulp was used to isolate volatile constituents. Prior to the isolation of aroma compounds, pitanga pulp was centrifuged at 1000 *g* for 5 min to remove particles. The supernatant was subjected to solvent-assisted flavor evaporation technique to split the pulp supernatant into the volatiles-containing aqueous and the non-volatiles-containing dry matter fraction [13].

### Identification of oxidoselina-1,3,7(11)-trien-8-one

After isolation of the volatile components from pitanga pulp by solvent-assisted flavor evaporation technique, the volatile-containing aqueous fraction was extracted five times with 70 mL of diethyl ether. The organic phases were combined and dried over anhydrous sodium sulfate. The solution was filtrated and concentrated using a rotary evaporator at 40°C and 450 mbar (Büchi, Flawil, Switzerland). The concentrated solution was adjusted to a final volume of 1.5 mL with diethyl ether. GC-MS analyses was performed on an Agilent 6890N GC system (Agilent Technologies, Böblingen, Germany) equipped with a DB-5MS column (30 m x 250 μm, film thickness 0.25 μm, Agilent Technologies, Vienna, Austria) connected to a 5973 mass selective detector (Agilent Technologies, Böblingen, Germany) that was operated in electron impact (EI) mode at 70 eV. Mass spectrum acquisition was performed in the mass range from m/z 35 to 300. A sample volume of 1 μL was injected at 250°C using the pulsed splitless mode at 50°C column temperature. The oven was held at this temperature for 2 minutes and then raised to 280°C in 15 minutes. The carrier gas was helium, which was used at a constant flow rate of 1.1 mL/min. The main compound, oxidoselina-1,3,7(11)-trien-8-one, eluted after 13.5 min and was identified based on its mass spectrometry fragmentation pattern using the Wiley 2008 reference database. GC-MS spectrum of oxidoselina-1,3,7(11)-trien-8-one (M^+^ = 232.1, C_15_H_20_O_2_): m/z (relative intensity) = 232 (28), 217 (4), 150 (18), 135 (44), 122 (82), 96 (60), 95 (58), 79 (37), 68 (100), 67 (40). For structural elucidation of oxidoselina-1,3,7(11)-trien-8-one, the oil was dissolved in acetonitrile-d_3_ and NMR spectra were recorded on a Bruker Avance III 500 MHz instrument (Bruker, Karlsruhe, Germany) at 500.32 MHz at ambient temperature. ^1^H-NMR (500 MHz, (CD_3_)_2_SO): δ (ppm) 1.24 [s, 10-Me], 1.75 [d, 4-Me], 1.93 [d, 11-Me_2_], 2.21 [s, 9-H_2_], 2.50 [m, 5-H], 2.84 [d, 6-H_2_], 4.38 [d, 1-H], 6.08 [d, 2-H], 6.21 [s, 3-H]; ^13^C-NMR (500 MHz; (CD_3_)_2_SO): δ (ppm) 23.1 [C4-Me], 28.3 [C10-Me], 29.2 and 31.1 [C11-Me_2_], 32.8 [C6], 33.9 [C10], 50.4 [C5], 57.6 [C9], 111.0 [C1], 119.9 [C4], 124.1 [C11], 130.9 [C7], 137.9 [C2], 140.8 [C3], 206.9 [C8].

### Isolation of oxidoselina-1,3,7(11)-trien-8-one

For the isolation of oxidoselina-1,3,7(11)-trien-8-one, 100 g of pitanga pulp were extracted five times with 140 mL diethyl ether. The organic phases were combined, dried over anhydrous sodium sulfate and concentrated to a volume of approximately 1 mL using the rotary evaporator operated at 40°C and 450 mbar. The solvent was completely removed with nitrogen gas. The residue was dissolved in 0.5 mL of acetonitrile and injected (50 μL) on an HPLC system (Dionex Ultimate 3000, Thermo Scientific, Vienna, Austria), connected to a diode array detector (Thermo Scientific, Vienna, Austria). Chromatographic separation was achieved on a Luna C18 column (250 x 3 mm, 5 μm, 100 Å, Phenomenex, Aschaffenburg, Germany) at 25°C using a mobile phase consisting of acetonitrile and H_2_O with a flow rate of 0.5 mL/min. A linear gradient was programmed starting with an increase of acetonitrile from 3% to 40% within 25 min, followed by a further increase of acetonitrile to 100% for the next 5 min. Data were collected using the Chromeleon 6.8 software (Thermo Scientific, Vienna, Austria). The peak absorbance was monitored at 190 nm. Peaks were collected and identified by GC-MS, using the same conditions as described above. The peak, which eluted at 18.7 min in HPLC analyses, corresponded to oxidoselina-1,3,7(11)-trien-8-one by means of GC-MS and NMR. The complete eluent was dried with nitrogen gas and appeared as yellow oil.

### Quantification of oxidoselina-1,3,7(11)-trien-8-one

For quantification, 100 g of pitanga pulp were subjected to high vacuum transfer using the solvent-assisted flavor evaporation technique and the volatile fraction was extracted with diethyl ether as described above. Oxidoselina-1,3,7(11)-trien-8-one was quantified in pitanga pulp by means of an external five-point calibration curve prepared with the isolated compound in the range of 70–1000 μg/mL. The calibration curve was linear (R^2^ = 0.996) within the range. The method showed good precision (RSD ≤ 7%). The quantification was performed on a HPLC system connected to a UV detector (Prominence, Shimadzu, Vienna, Austria), using the same column, linear gradient and UV wavelength used for isolation. The compound of interest eluted at 18.3 min at a flow rate of 0.5 mL/min at 25°C after injection of 10 μL of sample. The LOD and the LOQ was calculated to be 20.1 ± 0.74 μg/mL and 66.9 ± 2.47 μg/mL, respectively, according to the approach described in the European Pharmacopoeia. The results were expressed as μg/mL of pulp.

### Statistics

The data are presented as mean values ± standard deviation. For quantitative analyses, at least three independent experiments were performed. Two technical replicates were carried out for each quantitative analysis.

## Results and Discussion

Some studies already aimed at identification of the main organic micronutrients of pitanga, such as vitamins, polyphenols and carotenoids [3,14,15,16,17,18]. Besides the non-volatile constituents, volatile compounds were characterized in pitanga fruit as well as pitanga leaf oil [19,20,21,22,23]. However, there is no literature on the identification of volatile and non-volatile constituents in pitanga pulp, which is used to produce pitanga juice the major commercial pitanga product. The current study aimed to identify the major volatile and non-volatile low-molecular-weight constituents of pitanga pulp, as the main ingredient of the tropical juice.

### Identification of cyanidin-3-glucoside in purple-fleshed pitanga pulp

**Fig 1:**
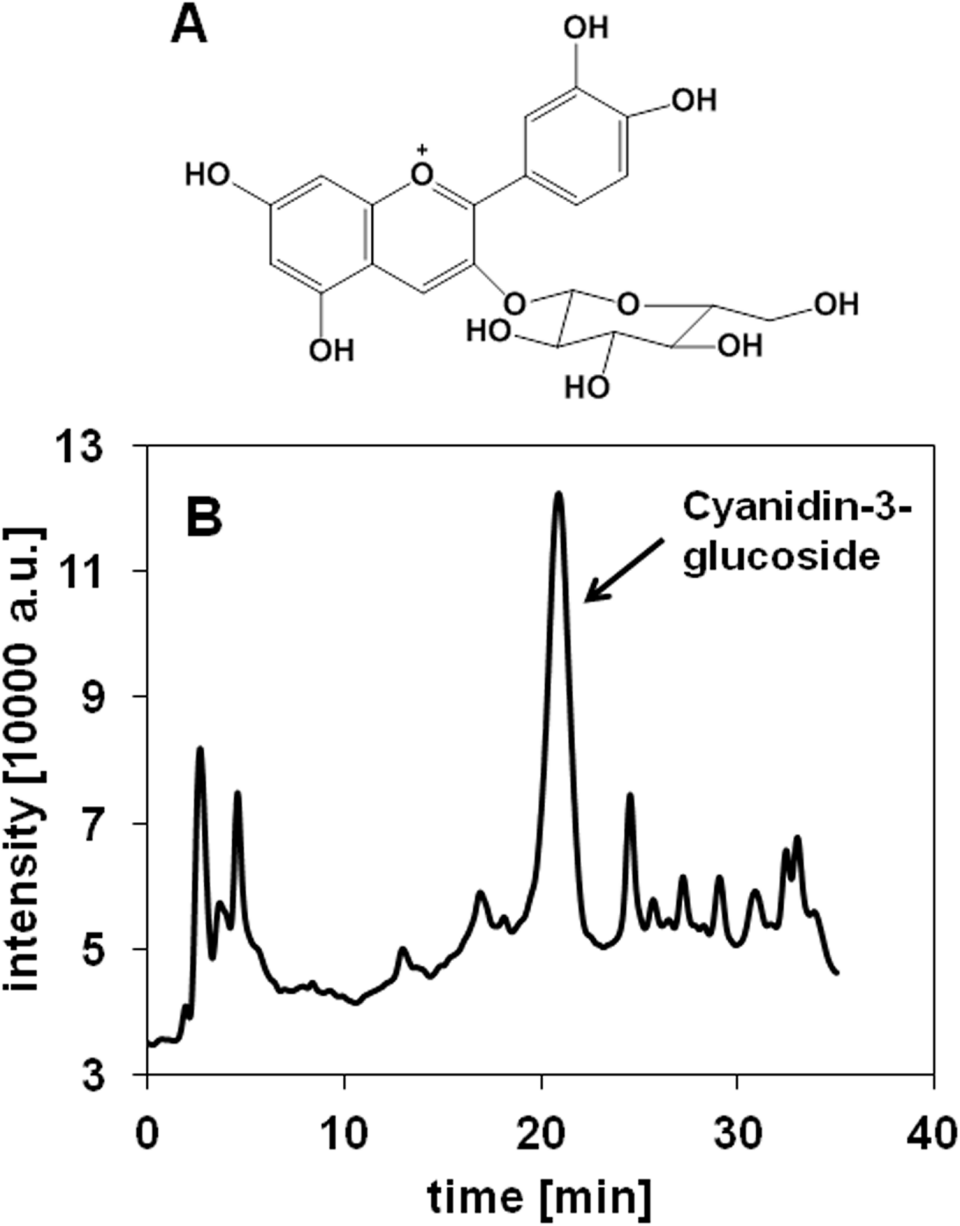
Identification of the non-volatile constituent, cyanidin-3-glucoside (A), in pitanga pulp. Total ion chromatogram of the pitanga pulp extract recorded in the LC-ESI (+) mode from m/z 100–1000 (B). Cyanidin-3-glucoside eluted at 20.8 min.

Cyanidin-3-glucoside was determined in pitanga pulp achieving 53.8% of the sum of intensities of all ions detected by LC-MS operated in the positive mode. The total ion chromatogram shows a distinct peak corresponding to cyanidin-3-glucoside (Fig 1). Identification of cyanidin-3-glucoside was confirmed by fragmentation of the precursor ion m/z 449 yielding the fragment m/z 287 (data not shown), which corresponds to that of cyanidin. Applying an external calibration method, cyanidin-3-glucoside, which eluted from the LC-column at 20.8 min, was quantified in pitanga pulp extract at a concentration of 344 ± 66.4 μg/mL. As the pitanga pulp extract was prepared by mixing 5 g dried pulp with 10 mL solvent, the concentration of the extract corresponds to 688 ± 132.8 μg/g dried pulp. The pitanga fruit consists of 77% pitanga pulp leading to an estimated concentration of the cyanidin-3-glucoside of 530 ± 102 μg/g fruit.

Cyanidin-3-glucoside has previously been quantified in pitanga pulp by Celli *et al*. [3]. These authors quantified cyanidin-3-glucoside in the pulp from red and purple varieties at different developmental stages. While there were either no or 7.45 ± 0.01 μg/g agylcone equivalents quantified in the pulp of the green (immature) pitanga fruit in the red and purple variety, respectively, the pulp at the red (mature) stage contained 31.0 ± 1.28 μg/g and 345 ± 2.37 μg/g cyanidin-3-glucoside in the red and purple variety, respectively. The pulp of a purple (overripe) fruit from the purple variety contained even 1690 ± 36.6 μg/g cyanidin-3-glucoside. These results indicate that the content of cyanidin-3-glucoside in the pitanga fruit depends on the variety as well as on the developmental stage. In the present study, the concentration of cyanidin-3-glucoside was determined in the pulp of the red fruit from the purple variety reaching approximately 700 μg/g dried pulp, which is comparable to the results obtained from the mature pitanga fruit by Celli *et al*. [3]. Cyanidin-3-glucoside was determined to be the predominant flavonoid and the main pigment responsible for the purple color of the pulp from the purple variety. In this study by Celli *et al*. [3], cyanidin-3-glucoside accounted for 59% of the total flavonoid glycosides in pitanga from the purple variety. However, the present study could even demonstrate that cyanidin-3-glucoside is the predominant non-volatile low-molecular-weight compound of the purple-fleshed pitanga pulp.

Recently, treatment of human gingival fibroblasts with juice-representative concentration of cyanidin-3-glucoside resulted in a 52 ± 9.9% inhibition of lipopolysaccharides-stimulated release of interleukin-8 [11]. Thus, the non-volatile constituent of pitanga pulp, cyanidin-3-glucoside, exhibited anti-inflammatory effects.

### Identification of the main volatile constituent of purple-fleshed pitanga pulp

To identify the main volatile compounds of purple-fleshed pitanga pulp, the pitanga pulp was fractionated into a volatile (aqueous distillate) and a non-volatile (dry matter) fraction using the solvent-assisted flavor evaporation technique [13]. This technique is based on a distillation unit connected to a vacuum pump, equipped with a nitrogen trap which isolates volatiles from complex food matrices, such as fruit pulps. After vacuum distillation, the yields of the aqueous distillate and the dry matter fractions, obtained from 100 g of fresh pitanga pulp, were 87.6 ± 2.38 g and 10.0 ± 1.01 g, respectively.

**Fig 2:**
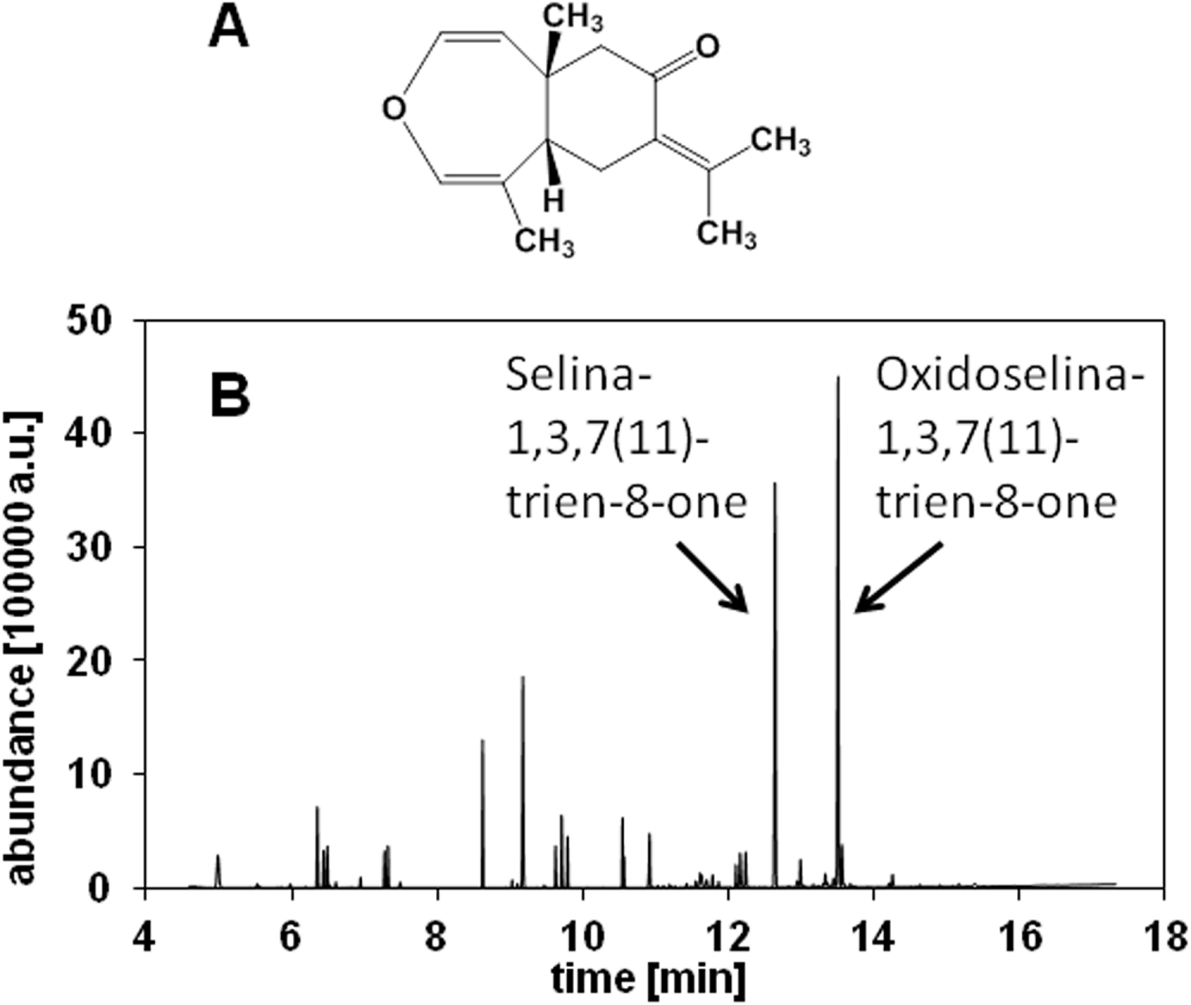
Identification of the most abundant volatile constituent, oxidoselina-1,3,7(11)-trien-8-one (A), in pitanga pulp. Total ion chromatogram of the volatile-containing fraction of the pitanga pulp recorded by GC-MS in the range of m/z 35 to 300 (B).

**Fig 3:**
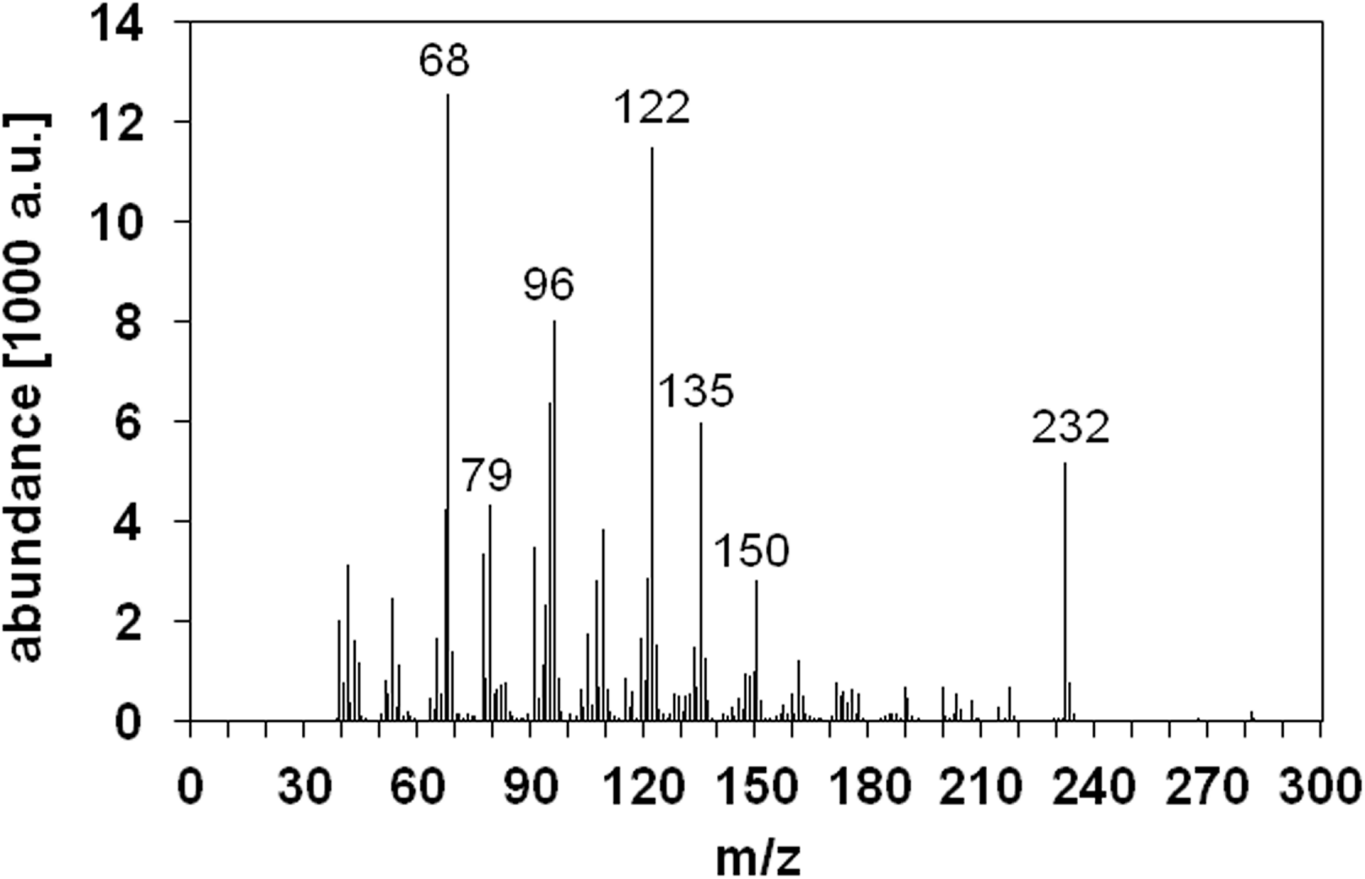
GC-MS spectrum of oxidoselina-1,3,7(11)-trien-8-one.

The main volatile constituent of the pitanga pulp was identified as oxidoselina-1,3,7(11)-trien-8-one (Fig 2). The total ion chromatogram revealed two main peaks corresponding to selina-1,3,7(11)-trien-8-one and oxidoselina-1,3,7(11)-trien-8-one with the latter being the most abundant volatile (Fig 2B). Fig 3 illustrates the EI-mass spectrum of oxidoselina-1,3,7(11)-trien-8-one, which showed a mass spectral similarity index of 95% when comparing it to the mass spectrum in the Wiley reference database.

Weyerstahl *et al*. [22] identified oxidoselina-1,3,7(11)-trien-8-one in pitanga leaf oil with a mass spectrum and ^1^H- and ^13^C-NMR spectrum comparable to that obtained in the current study. The oxepine skeleton of the ring A is indicated by the typical downfield and upfield shifts of α-C, H atoms and ß-C, H atoms, respectively, in the NMR spectra recorded at 105 K, thereby not favoring a 1,3-cyclohexadiene structure for ring A [22]. Other studies also reported the detection of selina-1,3,7(11)-trien-8-one in essential oils of pitanga leaves by GC-MS [20,21,24,25]. Besides the pitanga leaf extract, pitanga fruit was also analyzed for its volatile compounds [19]. Oliveira *et al*. [19] identified selina-1,3,7(11)-trien-8-one as a minor constituent (0.8%) of pitanga fruit.

Although the authors were able to detect several peaks, none of the compounds was identified or quantified as oxidoselina-1,3,7(11)-trien-8-one. For the first time, the current study reported the identification of oxidoselina-1,3,7(11)-trien-8-one with a relative abundance of 27.7% of total ions as detected by GC-MS and selina-1,3,7(11)-trien-8-one with a relative abundance of 22.2% of total ions as the major volatile constituents of pitanga pulp from the purple variety.

To quantify the content of oxidoselina-1,3,7(11)-trien-8-one in pitanga pulp using an external calibration curve, the compound was isolated from the pulp. After isolation by HPLC, structural identity was confirmed by NMR spectroscopy and GC-MS (Fig 3). The hereby isolated oxidoselina-1,3,7(11)-trien-8-one, which is not commercially available, was used to quantify oxidoselina-1,3,7(11)-trien-8-one in the pitanga pulp. A concentration of 89.0 ± 16.9 μg/mL of oxidoselina-1,3,7(11)-trien-8-one could be detected in 1.5 mL of the ether extract after subjecting 100 g of pitanga pulp to the SAFE apparatus. Thus, in one gram fresh pulp from the purple variety 1.34 ± 0.25 μg oxidoselina-1,3,7(11)-trien-8-one could be determined. As pitanga fruit consists of approximately 77% pulp the concentration of oxidoselina-1,3,7(11)-trien-8-one in the fruit can be estimated to be 1.03 ± 0.19 μg/g fruit. There are no quantitative data in the literature with regard to the concentration of oxidoselina-1,3,7(11)-trien-8-one in pitanga.

Only semi-quantitative results, which were obtained by calculating the relative abundance in pitanga with GC-MS, were reported in the literature [4,22]. Ogunwande *et al*. [4] reported a relative abundance of 11.0% for oxidoselina-1,3,7(11)-trien-8-one, which was identified as the fourth most abundant volatile compound in pitanga fruit. Similar results were obtained by Weyerstahl *et al*. [22] who determined a relative abundance of 14% for oxidoselina-1,3,7(11)-trien-8-one in pitanga leaf oil. In pitanga pulp from the purple variety, the present study demonstrated, for the first time, a relative abundance of 27.7% for oxidoselina-1,3,7(11)-trien-8-one, which corresponded to a concentration of 89.0 ± 16.9 μg/mL (1.34 ± 0.25 μg/g fresh pulp), identifying oxidoselina-1,3,7(11)-trien-8-one as the most abundant volatile constituent in pitanga pulp.

Taken together, the current study identified and quantified the main volatile and non-volatile low-molecular-weight constituents in the pulp of pitanga from the purple variety. As the pulp of pitanga is used to produce the widely consumed tropical pitanga juice, the identification and quantification of the major constituents in the pulp is of great importance.

The non-volatile constituent of pitanga pulp, cyanidin-3-glucoside, was shown to reduce cytokine-induced inflammation at a concentration of 11.2 μg/mL in intestinal cells by a 65% inhibition of prostaglandin E_2_ production [26]. The concentration of cyanidin-3-glucoside in pitanga pulp, as determined in the current study, is approximately 30-fold higher than the concentration of cyanidin-3-glucoside used by the study of Serra *et al*. [26], showing anti-inflammatory effects for cyanidin-3-glucoside.

For oxidoselina-1,3,7(11)-trien-8-one, anti-inflammatory activity could be demonstrated in lipopolysaccharides-stimulated human gingival fibroblasts by reducing the release of interleukin-8 by 45 ± 3.7% after treatment with oxidoselina-1,3,7(11)-trien-8-one in juice-representative concentration [11]. Thus, pitanga pulp and juice might also exert anti-inflammatory activities. Recently, an anti-inflammatory activity of pitanga juice on human gingival epithelial cells could be demonstrated by an attenuation of interleukin-8 release by 55 ± 8.2% and 52 ± 11% in non-stimulated and lipopolysaccharides-stimulated cells, respectively [11]. Future studies should investigate further biological effects of pitanga, pitanga juice and their major constituents, as identified and quantified in the current study, in juice-representative concentrations.
